# Shedding Light on Local Organizational Principles in the Primary Sensory Cortex

**DOI:** 10.1371/journal.pbio.0040420

**Published:** 2006-11-21

**Authors:** Jami Milton Dantzker

Humans see the world in rich color and detail. Rodents supplement their vision with long whiskers that provide tactile information about their environment. Neuroscientists can investigate how these senses are represented in the brain by identifying general organizational principles of the underlying neural circuits; different parts of the body surface have been mapped to specific brain areas. However, the resolution of this topographical brain map is coarse. It is analogous to a world map that shows the highways connecting major cities, but without details about local streets in each region. Tapping into the finer details of local information flow is a daunting task, because each cubic centimeter of the brain contains millions of neurons connected by billions of synapses.

Now, Ingrid Bureau, Karel Svoboda, and colleagues have shed light on the complexities of brain form at high resolution, revealing what may be a general principle by which sensory information enters and segregates in the primary sensory areas of the cerebral cortex. Sensory information is known to relay through a midbrain structure called the thalamus before entering the cerebral cortex. In the visual system, distinct types of properties about objects in a visual scene—such as motion or object texture—travel in separate pathways through the thalamus. This segregation of object information is maintained in the local circuits of the primary visual cortex (the first cortical area that processes visual information) and continues to remain relatively segregated in higher visual cortical areas. Bureau et al. show that in the whisker system of rodents, the flow of object touch and motion-related information transduced by whisker stimulation bears a striking similarity to the visual system. The distinct types of information remain segregated when entering the barrel cortex (the first cortical area that processes whisker information), and this segregation is maintained as the excitation ascends within the barrel cortex.

To unlock the secrets of local brain organization in the mouse barrel cortex, the authors used a technique termed laser scanning photostimulation (LSPS) that produces a fine-scale quantitative spatial map of functional connectivity. Neurons in the cortex are known to communicate via chemical synaptic contacts, and LSPS produces a spatial map of neurons that form functional synaptic contacts with one another. The authors performed LSPS in “thalamocortical” brain slices that preserved both the connections from the thalamus into the barrel cortex and the local connections between the eight laminar subdivisions of the barrel cortex (layers 1, 2, 3, 4, 5A, 5B, 6A, and 6B). For whisker information, the thalamus functionally segregates between two main pathways: the paralemniscal pathway, which travels anatomically through the posterior nucleus (POm) of the thalamus and conveys sensor motion (“whisking”) signals, and the lemniscal pathway, which travels through the ventral posteromedial nucleus (VPM) and conveys contact (“touch”) signals and whisking signals.

**Figure:**
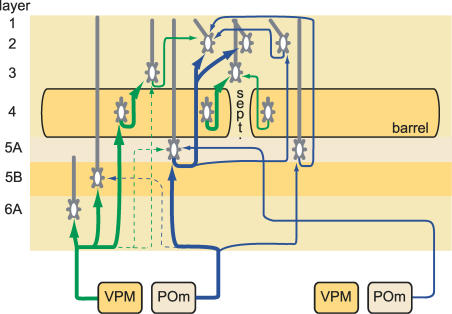
Circuit diagram of the thalamocortical and ascending intracortical projections in the barrel cortex. (Lemniscal projections, green; paralemniscal projections, blue.) Thick, thin, and dashed lines denote decreasing density of the projection.

Bureau et al. mapped connections from the lemniscal touch and paralemniscal whisking pathway into the barrel cortex and saw that these pathways connect into distinct layers in the barrel cortex. The lemniscal pathway makes direct connections primarily in three layers (layers 4, 5B, and 6), while the paralemniscal connects primarily in just one (layer 5A). Remarkably, layer 2/3 received little direct thalamic input from either pathway, a result that wasn’t easily predicted from simply following anatomical connections from the thalamus to the barrel cortex. However, when they continued to follow these pathways through the local circuits of the cortex, the authors saw that lemniscal layer 4 strongly targeted layer 3, and in contrast, paralemniscal layer 5A strongly targeted layer 2, with some diffuse connectivity in layer 3.

These findings reveal an unprecedented level of local functional organization and show for the first time that distinct types of information gathered by whisker stimulation about surrounding objects remain segregated both in the thalamus and in the barrel cortex. Future work will determine how these local pathways organize globally throughout higher cortical areas. Most importantly, with a better understanding of local and global organizational patterns in sensory areas, neuroscientists are one step closer to translating brain form into its exquisite function of accurately representing the world.

